# Treatment of Urticaria caused by severe cryptosporidiosis in a 17-month-old child – a case report

**DOI:** 10.1186/s12879-023-08446-y

**Published:** 2023-07-10

**Authors:** Mehdi Azami, Saeid Amini Rarani, Fatemeh Kiani

**Affiliations:** 1 Skin Diseases and Leishmaniasis Research Center, Isfahan University of Medical sciences, Isfahan, Iran; 2 Basir Laboratory Research and Development Center, Basir Medical Diagnostic Laboratory, Isfahan, Iran; 3 Department of Medical Parasitology and Microbiology, Hojjatieh Medical Diagnostic Laboratory, Hojjatieh Hospital, Isfahan, Iran; 4 Nursing and Midwifery Care Research Center, Department of Operating Room, Isfahan University of Medical Sciences, Isfahan, Iran

**Keywords:** *Cryptosporidium*, Nitazoxanide, Parasites, Urticaria

## Abstract

**Background:**

*Cryptosporidium* is an intracellular protozoan that causes gastrointestinal symptoms in humans and animals. In immunocompromised patients and children under 5 years of age, the infection is severe and can be life-threatening due to severe diarrhea.

**Case presentation:**

We report a case of urticaria associated with *Cryptosporidium* in a 17-month-old female Iranian child. The patient had moderate diarrhea (> 3 loose, watery stools but not more than 10 diarrhea stools in a day), weight loss, and acute urticarial (rash clears completely within 6 weeks). Since the child’s father worked in livestock farming, the parasite may have been transferred from the cow or calve to the house and the child. Several *Cryptosporidium* oocysts were detected in the modified acid-fast staining of the child’s stool sample. The patient was successfully treated with nitazoxanide (100 mg twice daily) and became negative for parasites three days after treatment and one week after discharge from the hospital. The child was observed to produce < 3 loose stools in the previous 24 h after 1-week post-treatment and after 6 months of follow-up.

**Conclusion:**

A number of parasites are associated with urticaria, but to our knowledge, there is no information on *Cryptosporidium*-induced urticaria. Therefore, our result may be evidence for the role of this parasite in the development of urticaria if other causes such as food allergies, autoimmune diseases and etc. don’t role in urticaria.

## Background

Cryptosporidiosis is one of the protozoan diseases caused by coccidia of the genus *Cryptosporidium*. Tyzzer first reported this protozoan in 1907 from the stomach glands of a laboratory mouse. *Cryptosporidium* is one of the most important common intestinal pathogens that has been reported in many organisms and in most parts of the world. The zoonotic role of *Cryptosporidium* has also made it important in terms of economics and public health [1].

This disease has been a serious problem in public health and at present it is widely considered as one of the causes of acute infectious gastroenteritis that is self-limiting in individuals with a complete immune system and fatal infection in immunocompromised individuals.

About 30 to 50% of the deaths of children and infants worldwide are due to cryptosporidiosis [2]. According to the World Health Organization (WHO) report, about 8 million deaths of children under 5 years of age are related to diarrhea [3]. In industrialized and developing countries, the prevalence rate of cryptosporidiosis ranges from 0.1 to 9.1% [4] and 2.98–25.9%, respectively [5]. In Iran, the range of cryptosporidiosis is 0.3–21.4% in children [6, 7] to 26.7% in immunosuppressive patients [8]. The prevalence of this protozoan in children with diarrhea in the neighboring countries was found to be 8.8% in Iraq, 16.6–27.9% in Egypt, 10.3% in Pakistan, and 7.3% in India [9]. The routes of *Cryptosporidium* spp. transmission are waterborne, food-borne, and occasionally person-to-person. Parasites cause weight loss, stunted growth, permanent impact on children’s development and increase in mortality. Acute cryptosporidiosis occurs in the form of severe watery diarrhea, which leads to dehydration, malabsorption and malnutrition. There are various reports of the prevalence of cryptosporidiosis in children in Iran. Previous studies have reported 4.6% prevalence of parasites in children under five years of age in Isfahan city [10].

The clinical symptoms of cryptosporidiosis are similar in children and adults. These symptoms include watery diarrhea that is sometimes with mucus. Diarrhea may be accompanied by fever, weight loss, nausea, vomiting, and loss of appetite. In severe cases, the parasite can attack the respiratory system, liver, bile ducts, and pancreas [1]. At present, the effective treatment for cryptosporidiosis is the use of nitazoxanide along with serum therapy.

Urticaria refers to localized or widespread red edematous papules on the body surface that may last for several weeks. Skin lesions may be accompanied by angioedema, which is characterized by pathological changes in the depth of the dermis and subcutaneous tissue [11]. Inflammatory mediators released from basophils and mast cells lead to an increase in these skin reactions. About 20% of the general population will develop urticaria at least once in their lifetime. Diagnosing urticaria is easy, but finding its cause is difficult and requires careful examination methods to discover diagnostic clues and rule out serious medical conditions.

Skin manifestations and urticaria have been reported by some helminth and protozoan parasites [12]. But so far, there is no report of *cryptosporidium* parasite skin manifestations. We describe a case of chronic urticaria associated with *Cryptosporidium*.

## Case presentation

A 17-month-old female referred to the pediatric clinic of Isfahan Hojjatiyeh hospital due to severe diarrhea with urticaria. She was born in the city of Isfahan and her parents lived in one of the villages around Isfahan. The child’s father was a farmer and her mother was a housewife. No similar clinical symptoms were observed in the child’s parents. The parents complained about the malaise of their child’s, abdominal cramps and diarrhea, which was accompanied by a red rash on the skin. The parents mentioned that the child’s symptoms started 20 days ago and visiting different doctors, taking antibiotics and prescribed medicines during this period were not effective. The child passed abnormal watery mucous and non-bloody loose stools 5 to 6 times a day. He was examined with moderate dehydration with minor symptoms due to dehydration such as weight loss, deep-set eyes, and pale and lethargic skin. Physical examination of the chest, abdomen, ears and throat was normal, but typical erythematous wheals accompanied by itching and without angioedema were observed on skin. The child had no fever, but presented tachycardia of 127 bpm, a respiratory rate of 20/min, and blood pressure of 85/6.0 mmHg. O_2_ saturation on room air was 99%.

**Fig. 1:**
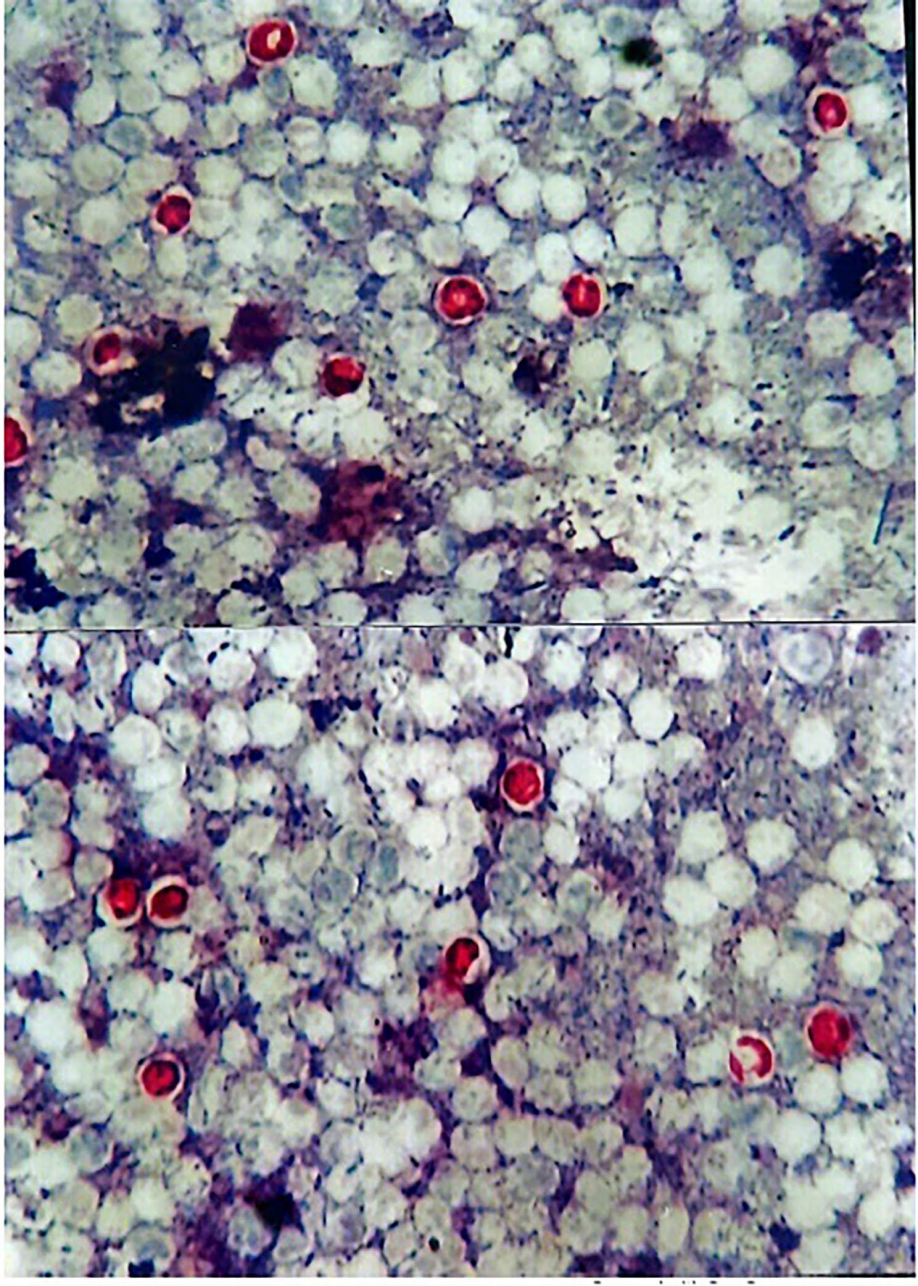
Microscopical analysis with Ziehl-Neelsen modified staining revealed numerous oocysts of *Cryptosporidium* spp. in the patient’s feces

Her peripheral blood count showed a white blood cell (WBC) count of 11.7 × 10^3^/mm3, a red blood cell count (RBC) of 4.6 × 10^6^/mm^3^, hemoglobin of 13.4 g/dL, hematocrit of 41.2%, reticulocytes 1.1% and a platelet count of 541 × 10^3^/ mm^3^. A differential count of WBC revealed the following: neutrophils 49%, lymphocytes 41%, eosinophils 6% and monocytes 4%. The results of biochemical tests were normal. Only a slight amount of hyponatremia (Na: 130.1 mmol/L) and hypokalemia (K: 3.0 mmol/L) was observed. The results of C-reactive protein, erythrocyte sedimentation rate, urine analysis and urine culture, thyroid function tests and thyroid autoantibodies, complement factors C3, C4 and C1 inhibitor, antinuclear autoantibodies (ANA, ENA), serum immunoglobulins, and rheumatoid factor were normal. Slightly IgE level was increased (19 IU/mL, Normal: 0–13 IU/mL). The patient’s stool sample was sent to the laboratory for rotavirus-adenovirus antigens (Immunochromatography rapid strip test, AllTest, China), *Giardia* antigen (Immunochromatography rapid strip test, VECHEK, China), *Clostridium* antigens (*Clostridium difficile* GDH + Toxin A + B, Immunochromatography rapid test, AllTest, China) and fecal bacteria culture (Xylose Lysine Deoxycholate Agar, HiMedia, India) and the all results were negative. Routine stool examination for enteric parasites was negative. Modified Ziehl-Nielsen staining of a stool smear showed several *Cryptosporidium* oocysts (Fig. 1). Intravenous fluids with oral paromomycin (30 mg/kg/day) were started for the patient. With the diagnosis of *Cryptosporidium*, nitazoxanide oral solution (100 mg twice daily) was included in the patient’s medication regimen. One day after taking the medicine, the severity of diarrhea decreased, so that after 3 days, the diarrhea was completely cured. At the same time as the frequency of diarrhea decreased, the severity of urticaria also decreased and after 5 days it was generally improved. One week after taking nitazoxanide, the patient was discharged from the hospital with a good general condition.

By Modified Ziehl-Nielsen staining method, the presence of *Cryptosporidium* in the feces was reported negative three days after taking the drug and one week after being discharged from the hospital. Her stool pattern normalized in 1 week and she has had no recurrence after 6 months of follow-up.

## Discussion

*Cryptosporidium* is a zoonosis and intestinal pathogen that infects humans and various animals. The parasite is transmitted through water, food, and feces-contaminated materials, and settles in the epithelial cells of the intestinal region where it reproduces asexually and increases in number. The presence of parasites in the intestine cause’s mild to severe gastrointestinal symptoms. The parasite causes severe watery diarrhea that can lead to death in immunocompromised patients and children if left untreated [13]. A recent study showed, zoonosis transmission of *Cryptosporidium* species due to contact with cows and calves is prevalent among farm workers and their household members in Isfahan city [14]. According to the results of this study, most of the people infected with parasites were without diarrhea or clinical symptoms. It is worth discussing this as it probably represents a further risk factor for transmission especially if this was shown to be in young children from which transmission is likely to occur [14]. In our case, the father of the family was in close contact with cows and calves, and perhaps this factor caused the parasite to be transmitted to the child.

Several conditions have been found to be related to urticaria symptoms, whereas the term chronic idiopathic urticaria identifies the high percent of conditions in which a pathogenetic factor has not been found [15]. Identifying the cause of urticaria requires ruling out many other conditions. A special form of urticaria is autoimmune urticaria, in which antibodies are produced against IgE receptors or IgE itself [16]. Food allergens, drugs, colors, perfumes, insect bites, chemicals and physical sensitivities are other causes of urticaria. The existence of some diseases related to the immune system, viral and bacterial infections are also other causes of urticaria. Although intestinal parasites have been cited as a possible cause of urticaria, only a limited number of documented cases have shown a relationship between parasitic infection and urticaria [17]. A large number of infections caused by parasitic worms such as *Fasciola hepatica*, *Strongyloides stercoralis*, *Echinococcus granulosus*, filaria, *Ascaris lumbricoides*, *Trichinella spiralis*, and *Schistosoma* sp. have been associated with allergic skin symptoms, and antiparasitic drugs have not been effective in treating urticaria caused by them [12, 17, 18].

The mechanism of urticaria caused by Cryptosporidium, like other enteric pathogens, is not fully understood. The authors hypothesized a pathogenesis similar to that underlying the IgE-mediated allergic response: Cryptosporidium antigens induce specific Th2 clones on lymphocytes, which in turn produce specific cytokines (ILs 3, 4, 5 and 13) and lead to the switch of antibodies and IgE production. This mechanism can be confirmed by performing a skin biopsy and observing the increase in mast cells and mononuclear cells. This reaction will be responsible for increasing the production of histamine and other mediators of early and late inflammatory reaction.

The increase of circulating eosinophils in most patients with parasitic infection is another factor related to allergic sensitivity [19].

A number of antibiotics have been used to treat intestinal cryptosporidiosis. However, most of them cannot kill the organism completely [20]. Paromomycin is an aminoglycoside that cannot be completely absorbed when taken orally, and as an anti-cryptosporidial drug, it has a high rate of recurrence after treatment [21]. Perhaps one of the reasons is that paromomycin is placed inside the intestinal tract and has little activity on the intracellular forms of the parasite. Nitazoxanide (Alinia) is a new drug with broad activity against protozoa and intestinal worms. This drug inhibits ferredoxin reductase, which is the main enzyme in the parasite’s anaerobic metabolism [22]. Nitazoxanide has recently been approved by the FDA for the treatment of *Giardia* and *Cryptosporidium* in immunocompetent children aged 1 to 12 years. Along with drug treatment, appropriate intravenous nutrition, supportive hydration is necessary due to severe diarrhea. In our case, complete improvement of urticaria was observed using nitazoxanide, a drug that has no anti-allergic and anti-inflammatory effects, without taking any antihistamine. Unfortunately, we do not know the exact mechanism of urticaria caused by *Cryptosporidium*. Anyway, various reasons have been discussed for the cause of urticaria caused by parasitic infections, such as the role of specific IgE, Th2 cytokine skewing, eosinophil’s and coagulation system and finally circulating immune complexes and complement system [15–18].

## Conclusion

Our patient was a child with severe diarrhea with typical urticaria. Its cause was determined by an accidental intestinal parasitic infection with *Cryptosporidium*, which caused an allergic skin reaction. The treatment of parasitic infection led to the disappearance of parasite antigens in feces and the improvement of intestinal symptoms and urticaria. These results may provide evidence for the role of intestinal parasitic infections in the induction of chronic urticaria, especially by *Cryptosporidium* species.

## Data Availability

Not applicable.
